# Mice with Chimeric Livers Are an Improved Model for Human Lipoprotein Metabolism

**DOI:** 10.1371/journal.pone.0078550

**Published:** 2013-11-04

**Authors:** Ewa C. S. Ellis, Scott Nauglers, Paolo Parini, Lisa-Mari Mörk, Carl Jorns, Helen Zemack, Anita Lövgren Sandblom, Ingemar Björkhem, Bo-Göran Ericzon, Elizabeth M. Wilson, Stephen C. Strom, Markus Grompe

**Affiliations:** 1 Department of Clinical Science, Intervention and Technology (CLINTEC) Division of Transplantation Surgery, Karolinska Institute, Karolinska University Hospital Huddinge, Stockholm, Sweden; 2 Papé Family Pediatric Research Institute, Oregon Stem Cell Center, Oregon Health Science University, Portland, Oregon, United States of America; 3 Department of Laboratory Medicine, Karolinska University Hospital Huddinge, Stockholm, Sweden; 4 Yecuris Corporation, Portland, Oregon, United States of America; Laurentian University, Canada

## Abstract

**Objective:**

Rodents are poor model for human hyperlipidemias because total cholesterol and low density lipoprotein levels are very low on a normal diet. Lipoprotein metabolism is primarily regulated by hepatocytes and we therefore assessed whether chimeric mice extensively repopulated with human cells can model human lipid and bile acid metabolism.

**Design:**

FRG *[* ***F*** *ah(−/−)* ***R*** *ag2(−/−)Il2r* ***g*** *(−/−)]*) mice were repopulated with primary human hepatocytes. Serum lipoprotein lipid composition and distribution (VLDL, LDL, and HDL) was analyzed by size exclusion chromatography. Bile was analyzed by LC-MS or by GC-MS. RNA expression levels were measured by quantitative RT-PCR.

**Results:**

Chimeric mice displayed increased LDL and VLDL fractions and a lower HDL fraction compared to wild type, thus significantly shifting the ratio of LDL/HDL towards a human profile. Bile acid analysis revealed a human-like pattern with high amounts of cholic acid and deoxycholic acid (DCA). Control mice had only taurine-conjugated bile acids as expcted, but highly repopulated mice had glycine-conjugated cholic acid as found in human bile. RNA levels of human genes involved in bile acid synthesis including *CYP7A1*, and *CYP27A1* were significantly upregulated as compared to human control liver. However, administration of recombinant hFGF19 restored human *CYP7A1* levels to normal.

**Conclusion:**

Humanized-liver mice showed a typical human lipoprotein profile with LDL as the predominant lipoprotein fraction even on a normal diet. The bile acid profile confirmed presence of an intact enterohepatic circulation. Although bile acid synthesis was deregulated in this model, this could be fully normalized by FGF19 administration. Taken together these data indicate that chimeric FRG-mice are a useful new model for human lipoprotein and bile-acid metabolism.

## Introduction

The use of experimental animals in scientific research has enabled some of the most important breakthroughs in medical research [1]. Further refinement of animal models through genetic manipulations is an important and powerful tool in research today. Transplanting human cells and tissues into genetically engineered mice expands these possibilities. Humanized mouse models present opportunities to study whole cellular systems in an *in vivo* setting [2], [3], [4], [5].

Mice and human differ greatly in many aspects of cholesterol metabolism ranging from lipoprotein processing to cholesterol catabolism through bile acid synthesis. In mice, serum cholesterol is found mainly in high-density lipoproteins (HDL), while humans have mainly low-density lipoproteins (LDL). Several of the apolipoproteins synthesized by the liver are different in man and mice, such as ApoB and ApoE, and others such as Lp(a) are absent in mice altogether. Genetically modified mouse strains have been developed for atherosclerosis research, but the information gained has been limited because of the major species differences and the complex nature of cholesterol and lipid metabolism [6], [7], [8]. Furthermore catabolism of cholesterol via bile acid synthesis differs in mice and humans. Mice have an additional bile acid, muricholic acid, not present in humans, with beta-muricholic acid as the major form. It is well known that the different bile acids regulate overall bile acid synthesis differently in different species [9]. Regulation of the rate limiting enzyme in bile acids synthesis, cholesterol 7alpha-hydroxylase is dissimilar, and frequently opposite in rodents and man [10]. The murine promoter of this gene has a response element for LXR which is not present in humans [11]. Thus, stimulation of LXR by cholesterol leads to a feed-forward regulation that increases the synthesis of bile acids in mice, but not in humans.

Endocrine signaling between intestine and liver differ in man and mice. Humans secrete fibroblast growth factor 19 (FGF19) in response to increases in the ileal bile acid pool that results in a down-regulation of hepatic *CYP7A1*, the rate-limiting enzyme in bile acid synthesis. In contrast, mouse intestine signals through FGF15 [12], [13].

There are also species differences in conjugation of bile acids. Humans can amidate bile acids with both glycine and taurine [14], with a preference for glycine in adulthood. Mice conjugate almost exclusively with taurine [15].

Given the number of differences between mouse and human cholesterol and bile acid regulation and profiles, and considering that the liver is the major organ involved in the synthesis of these proteins, a mouse model with livers repopulated with human hepatocytes offers a useful model to investigate these pathways, *in vivo*. The aims of this study were to determine whether cholesterol and bile acid metabolism in FRG mice repopulated with human hepatocytes displayed a characteristic human profile, composition and regulation.

## Methods

Human liver tissue and hepatocytes were obtained through the Liver Tissue Cell Distribution System, and the studies were exempted by IRB 0411142 since no human subjects were involved (University of Pittsburgh). All animal work was conducted according to approved Institutional Animal Care and Use Committee (IACUC, Yecuris) protocol DN000024 and NIH OLAW assurance #A4664-01. The protocols follow the NIH guidelines for laboratory animal use and welfare.

### Transplantation of FRG mice

FRG mice were maintained as described previously [16]. Mice are maintained on NTBC (Nitisinone, Swedish Orphan International, Stockholm) in the drinking water (16 mg/l). Mice are injected, IP, 24 hr prior to transplant with 10^9^pfu of an adenoviral vector expressing the secreted form of uPA and receive up to 1 million human hepatocytes in 100 microliters of DMEM media via splenic injection. Following transplant, NTBC is gradually withdrawn to initiate loss of native hepatocytes. Progress of humanization is monitored monthly blood analysis by ELISA assay for human serum albumin (hSA). In general 1 mg/ml of circulating hSA correlates with ∼20% engraftment of human cells, 2 mg with ∼40%, and animals with 4 mg are approximately 80% repopulated. Hepatocytes were obtained from the Liver Tissue and Cell Distribution System, University of Pittsburgh or commercially available sources. Human hepatocytes (fresh and from serial transplantation) were cold-stored in University of Wisconsin solution for up to 48 hours, allowing additional time for transplants. Serial transplants were conducted as described previously [16]. At the time of serial transplantation, an aliquot of the cells were used for RNA isolation and the rest for transplantation.

At sacrifice, liver tissues was collected and snap frozen in liquid nitrogen for RNA expression analysis, serum was collected for measurement of lipoproteins and bile acid intermediates and gallbladder bile was collected for bile acid analysis.

### Lipid analysis

Cholesterol content of serum lipoproteins was separated by size exclusion chromatography from mouse or human serum and was measured according to Parini et al [17].

### Western blotting of mouse and human Apo E

Serum samples were separated by electrophoresis on 10% Bis-TrisNuPAGE Gel (Invitrogen). Proteins were transferred to a nitrocellulose membrane (Invitrogen) and incubated with rabbit anti human ApoE (Gene Tex GTX 101456) or rabbit anti mouse ApoE (Pierce PAI-46367). Donkey anti-rabbit HRP-conjugated IgG (GE Healthcare) was used as the secondary antibody. Signal was detected using the ECL kit according to instructions (Thermo Scientific).

### GC-MS analysis of bile acids in bile

Bile acids were analyzed as previously described by Björkhem et al [18] and Ellis et al.[10]. Briefly, 10 ul of gallbladder bile was diluted with 1 ml of water, 2 ml of 50% EtOH, 1g KOH and hydrolyzed together with 2500 ng deuterium labeled Cholic acid (D_5_) and chenodeoxycholic acid (D_4_), Deoxycholic acid (D_4_), Ursodeoxycholic acid (D_4_) at 125° C over night. Samples were diluted with saline and extracted twice with ether to remove neutral steroids. Following acidification with HCl (6M) to pH 1, bile acids were extracted with ether. The ether phase was methylated with trimethylsilyldiazomethane (Sigma cat.:36,283-2) and silylated using hexamethyl-disilazane (Alfa Aesar L16519) and trimethylchlorosilane (Merck 1.02333.0100) in pyridine at 60° C for 30 minutes. Solvent was evaporated and the samples dissolved in 200 ul of Hexane and analyzed by GC-MS (Agilent 5973 6890N). Data were analyzed using Agilent Mass hunter software.

### LC-MS/MS analysis of bile acid conjugates in bile

Bile acids were analyzed using HPLC-MSMS using a modified method initially described by D Tagliacozzi et al. [19]. Two ul of bile was mixed with 800ng internal standards in 40 µl methanol and 800 ul acetonitrile. The mixture was centrifuged at 13 000 x g for 15 minutes and the upper phase was transferred to a disposable glass centrifuge tube and evaporated under N_2_. Residue was dissolved in 75 ul of Methanol, vortexed and transferred to Waters vials. Tubes were rinsed with 75 ul 40% Methanol in water, 0.02% Formic acid and 10 mM Ammonium acetate and pooled. A Waters LC-MS/MS MicromassQuattro Micro, equipped with a C18 reverse- phase column and ESI in negative mode was used for analysis.

Six different deuterium labeled internal standards (D_5_-CA, D_4_-UDCA, D_4_-LCA, D_4_- GCA, D_4_-GUDCA, D_4_-GLCA), and unlabeled unconjugated bile acids (LCA, DCA, CDCA, HDCA, UDCA, CA, HCA, BMCA, AMCA and OMCA) and glycine- as well as taurine- conjugated bile acids (GLCA, GDCA, GCDCA, GCA, GUDCA, TLCA, TDCA, TCDCA, TCA, TUDCA) were used for calibration and quantification.

Unconjugated bile acids were measured by molecular anions (no product ions are produced). Glycine- or taurine-conjugated bile acids were quantified from negative daughter ions, generated after loss of the conjugate.

### FGF19 administration

Twelve FRGN mice were used, six were repopulated with human hepatocytes and six were used as controls. When serum human albumin levels indicated the mice were repopulated with human hepatocytes, FGF19 was administered. Recombinant human FGF-19 (PeproTech, Catalog # 100-32) was reconstituted in 0.9% saline with 0.1% BSA and three humanized and three control FRGN mice were injected (s.q.) with 0.5 mg/kg FGF19 twice daily for three days. Three humanized and three control FRGN mice were injected with diluents only. Mice were killed between 1–3 hours after the final injection, after their gallbladders had been cannulated for a 15–20 minute collection of bile. Serum and liver were harvested and snap frozen in liquid nitrogen.

### RNA

RNA was extracted using Trizol (Invitrogen cat#: 15596-026). Integrity was checked on a 1% agarose gel with 1xTAE and concentration measured using the Nano Drop (ND-1000) spectrophotometer.

### CDNA synthesis

A high capacity cDNA reverse transcription kit from Applied Biosystems cat# 4374966 with RNAse inhibitor was used according to instructions.

### QPCR

RNA expression was quantified using real time PCR (ABI prism 7000). For human genes predesigned Taqman probes were used. hCyp8B1: Hs00244754_s1, hCyp27A1: Hs00168003_m1, hCyp7A1: Hs00167982_m1, hCyc (PPIA): Hs99999904_m1, hSHP: Hs00222677_m1, hFGF19: Hs 00192780_m1, hABCB11: HS00184824_m1, hNTCP: HS00161820_m1, hFXR: Hs00231968_m1.

For mouse genes the SYBR Green method was used with the following primer sequences;mCyclophilinFw: GAT-GAG-AAC-TTC-ATC-CTA-AAG-CAT-ACA, mCyclophilin Rev: TCA-GTC-TTG-GCA-GTG-CAG-ATA-AA, mCYP7A1 Fw: AGC—AAC-TAA-ACA-ACC-TGC-CAG-TAC-TA, mCYP7A1 Rev: GTC-CGG-ATA-TTC-AAG-GAT-GCA, mGAPDHFw: TGT-GTC-CGT-CGT-GGA-TCT-GA, mGAPDH Rev: CCT-GCT-TCA-CCA-CCT-TCT-TGA-T, mABCG5 Fw: TGG-ATC-CAA-CAC-CTC-TAT-GCT-AAA, mABCG5 Rev: GGC-AGG-TTT-TCT-CGA-TGA–ACT-G, mABCG8 Fw: TGC-CCA-CCT-TCC-ACA-TGT-C, mABCG8 Rev: ATG-AAG-CCG-GCA-GTA-AGG-TAG-A, mSHP-Fw: AAG-GGC-ACG-ATC-CTC-TTC-AA, mSHP-Rev: CTG-TTG-CAG-GTG-TGC-GAT-GT

### Statistics

Data were analyzed either by the non-parametric Mann-Whitney U test, the non-parametric Kruskal-Wallis test or by 1-way ANOVA followed by post-hoc comparison according to Dunett or to LSD tests. In order to stabilize variances, data were transformed prior to ANOVA.

## Results

### Serum Lipoprotein profiles

**Figure 1:**
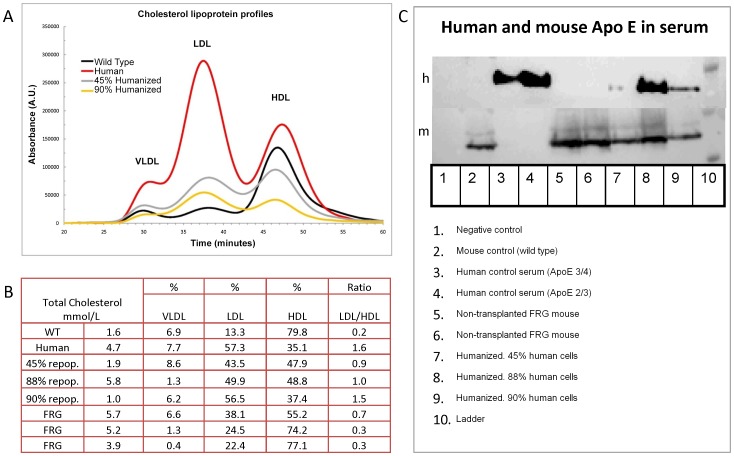
Lipoproteins in mouse serum. A, Serum cholesterol lipoprotein profiles measured by size exclusion chromatography of wild type mice, human, high (90%) and low (45%) levels of repopulation in humanized FRG mice. Panel B showing percentage of different lipoprotein fractions, as well as ratio of LDL/HDL in wild type mice, human controls, repopulated FRG mice and FRG controls. C, Western blot analysis of human (h) and mouse (m) Apolipoprotein E in serum samples of human and mouse control samples, 1–6. Humanized FRG with different levels of repopulation are shown in lane 7–9.

Cholesterol metabolism involves complex interplays between absorption, production and excretion. In mice, serum cholesterol is found mainly as high-density lipoproteins (HDL), while in man, low density lipoproteins (LDL) predominate. Since lipoprotein synthesis is a hepatic function, in mice with livers repopulated with human hepatocytes, one could expect serum lipoproteins to change from a HDL to an LDL centered profile. Cholesterol lipoprotein profiles were measured in serum of wild type, non-repopulated and repopulated mice as well as a human control sample, figure 1A. Data presented in figure 1B shows the cholesterol content of different lipoprotein fractions. In wild type and non-repopulated FRG mice HDL is the predominant lipoprotein constituent. In human serum samples and in FRG mice repopulated with human hepatocytes, HDL was decreased while LDL was increased from a ratio of LDL/HDL of approximately 0.3 in non-repopulated animals to 0.9, 1.0, 1.5 in mice repopulated to 45, 88 or 90%, respectively, approaching the value of 1.6 from a healthy 38 year old female.

### Apolipoprotein E

Apolipoprotein E is synthesized by hepatocytes and also binds to hepatic receptors as part of the catabolic pathway for triglyceride-rich lipoproteins. Western blot analysis, shown in figure 1C, revealed that FRG mice repopulated with human hepatocytes synthesize and secrete human and mouse ApoE.

### Bile acid conjugates

Bile acids are conjugated in hepatocytes prior to excretion into bile. The conjugation of bile acids differs significantly between species; mice conjugate almost exclusively with taurine whereas humans conjugate with both glycine and taurine at a ratio of approximately 5:1. In mice repopulated with human hepatocytes one could expect to find glycine conjugated bile acids. Bile acids conjugates were analyzed in mouse bile using LC-MS/MS. Table 1 shows the percentages of taurine conjugated cholic acid (T-CA), glycine conjugate cholic acid (G-CA) and unconjugated cholic acid (CA) in humanized and control mice. The results showed that in highly repopulated mice (88–94% humanized) the proportion of T-CA was decreased and both free CA and G-CA increased relative to FRG controls.

**Table 1:** LC-MS/MS analysis of conjugates of cholic acid in gallbladder bile of control FRG mice and mice repopulated at different levels.

Mouse ID	Humanized	%T-CA	%G-CA	%CA	Ratio T-CA/G-CA
FRG 10	0%	99.8	0.17	0.02	587
FRG 1	0%	98.6	0.15	1.30	657
FRG 2	0%	99.4	0.11	0.52	903
TxFRG 2	94%	80.8	8.11	11.07	10
TxFRG 4	90%	81.5	6.96	11.53	12
TxFRG 5	88%	87.4	1.50	11.12	58
TxFRG 8	78%	99.4	0.47	0.08	211
TxFRG 11	45%	95.7	0.14	4.11	684

Data showing percentage of taurine conjugated cholic acid (T-CA). glycine conjugated.

cholic acid (G-CA) and free cholic acid (CA).

### Bile acid composition

**Figure 2:**
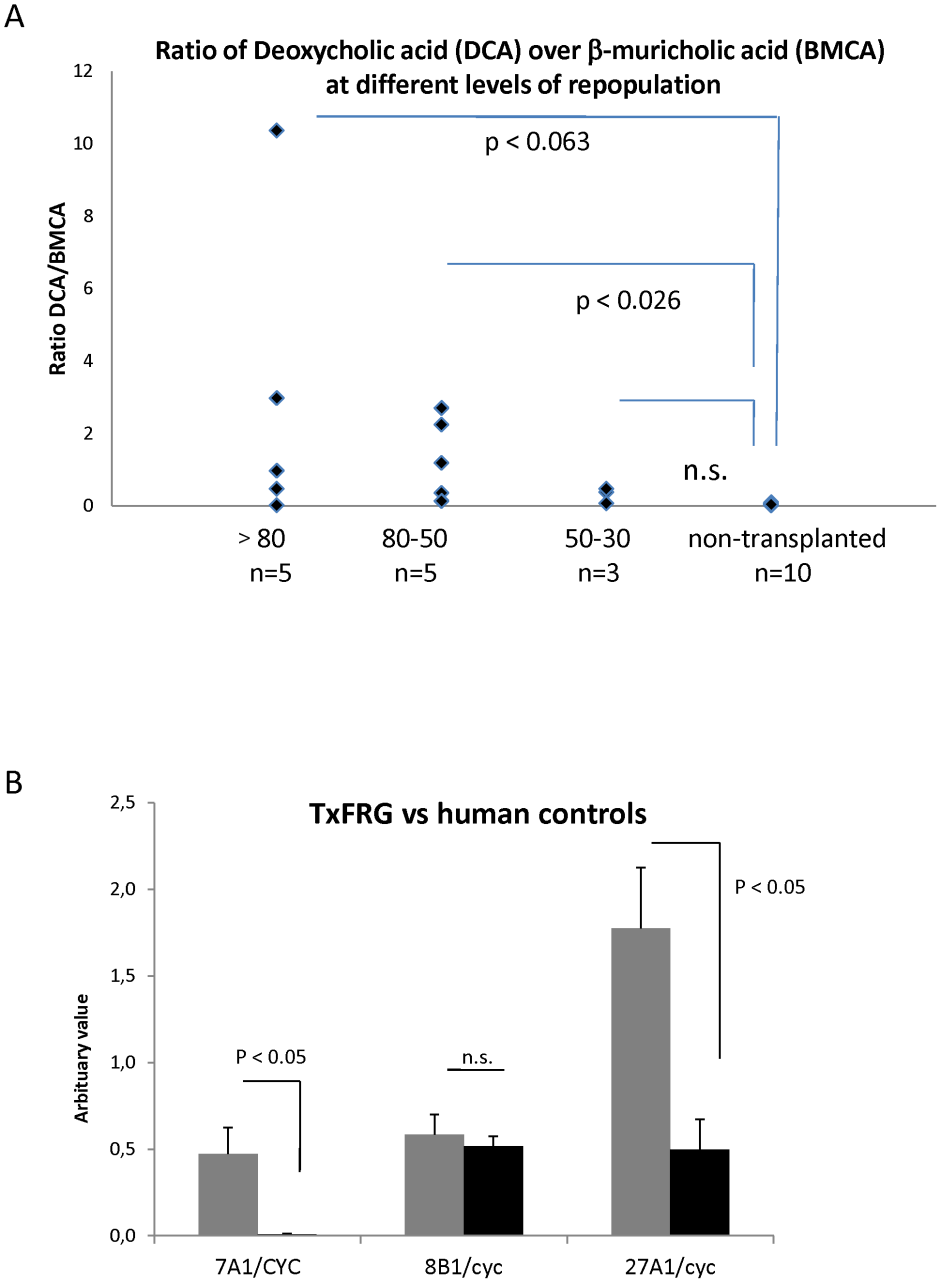
Bile acid composition and expression of enzymes of the bile acid synthesis pathway. A, Ratio of deoxycholic acid (DCA) over beta-muricholic acid (BMCA) in high (<80%), moderate (50–80%) or low (30–50%) population compared to non-transplanted FRG mice. Statistics were performed by a 1-way ANOVA on log-transformed data followed by Dunett’s test. The overall significance was p = 0.023 (all different vs control). B, RNA expression of *CYP7A1*, *CYP8B1* and *CYP27A1* in hepatocytes, normalized to cyclophillin analyzed by quantitative real time PCR. Expression of human genes were analyzed in hepatocytes isolated from humanized FRG (Tx-Mice) and compared to isolated human hepatocyte controls (Human). Statistics were performed by a non-parametric Mann-Whitney U test.

Bile acid composition in mice differs from humans by the presence of additional bile acids in mice, alpha, beta and omega- muricholic acid, with beta as the major form. In rodents bile acids that have been de-hydroxylated in the intestine producing the secondary bile acid deoxycholic acid (DCA) can be re-hydroxylated to cholic acid. Humans do not re-hydroxylate and therefore have higher levels of secondary bile acids such as DCA. Following hydrolysis, we extracted bile acids from 1 ul of bile and analyzed them by GC-MS. Table 2 shows percentage of individual bile acids and the ratio of DCA to beta-muricholic acid (BMCA). As shown in figure 2A, the ratio of DCA/BMCA from non-transplanted mice was significantly different in highly repopulated < 80%, (p = 0.063) and moderately repopulated mice (50–80%, p = 0.026). In mice with a low degree of repopulation (30-50%), the ratio of DCA/BMCA was not significantly different from non-transplanted animals.

**Table 2:** Bile acid composition (%) in gallbladder bile collected from control mice (FRG), n = 13 or humanized mice (TxFRG), n = 10.

Mouse ID	Level of hum.	DCA	CDCA	AMCA	CA	UDCA	HCA	BMCA	OMCA	DCA/BMCA
TxFRG1	90	73	4	0	14	2	0	7	0	10.36
TxFRG2	94	17	7	12	42	4	ND	18	ND	0.98
TxFRG3	86	1	3	6	47	1	0	35	7	0.02
TxFRG4	90	20	1	1	70	0	1	7	2	2.98
TxFRG5	88	5	4	5	74	1	0	11	0	0.47
TxFRG6	79	8	9	8	67	2	0	7	0	1.19
TxFRG7	78	13	1	0	79	0	0	6	1	2.25
TxFRG8	78	9	2	3	61	1	ND	25	ND	0.36
TxFRG9	70–80	3	2	3	61	1	0	19	11	0.14
TxFRG10	70–80	4	0	1	92	0	0	2	0	2.71
TxFRG11	45	10	5	3	54	2	ND	26	ND	0.38
TxFRG12	30	1	2	3	68	1	1	19	6	0.07
TxFRG13	30	8	2	9	60	3	ND	17	ND	0.47
FRG1	0	0	1	2	62	1	0	24	9	0.02
FRG2	0	0	1	3	59	1	1	15	21	0.03
FRG3	0	3	4	11	48	5	ND	29	ND	0.10
FRG4	0	2	5	8	56	2	ND	27	ND	0.06
FRG5	0	2	6	2	43	2	ND	45	ND	0.04
FRG6	0	2	16	0	36	1	ND	45	ND	0.04
FRG7	0	4	31	0	28	1	ND	36	ND	0.10
FRG8	0	2	6	10	57	4	ND	21	ND	0.08
FRG9	0	1	8	1	62	2	ND	26	ND	0.05
FRG10	0	2	7	10	31	6	ND	44	ND	0.05

### RNA expression in humanized mice

Expression of the rate limiting enzyme in the bile acid synthesis Cholesterol 7alfa-hydroxylase, *CYP7A1*, revealed a significant (p<0.05) increase from 0.008 (arbitrary value) in humans (n =  5) to 0.473 in humanized mice. This reflects a∼57-fold of *CYP7A1* increase in humanized mice (figure 2B).

The expression of Sterol 27-hydroxylase(*CYP27A1*), the enzyme responsible the first step in the side chain degradation and the first step of the acidic pathway of bile acid synthesis, was also significantly increased from 0.5 (arbitrary value) in humans (n =  5) to 1.8 in humanized mice (n =  3), p<0.05 (figure 2B).

The expression of Sterol 12α-hydroxylase (*CYP8B1*), the enzyme responsible for formation of cholic acid (and subsequently deoxycholic acid), was not significantly different in humanized mice (0.58) compared to human controls (0.52) (figure 2B).

### Administration of FGF19

We hypothesized that the 57-fold increase in *CYP7A1*was due to a mismatch in signaling between the murine intestine and human hepatocytes. We injected recombinant human FGF19, 0.5 mg/kg body weight, subcutaneously (s.q.) twice daily for 3 days into humanized (TxFRG) mice or non-humanized FRG controls. The experiment was terminated 5 hrs after the last injection, bile was collected over a 15 minute period and the liver snap frozen for RNA expression analysis.

Administration of FGF19 lead to a significant decrease of the total bile acid concentration in bile of humanized mice, from 24 500 ng/ul to 9 000 ng/ul, p = 0.001 (table 3). Non-transplanted mice injected with FGF19 also exhibited the same effect decreasing from 17 300 ng/ul to 9 450 ng/ul after infusion (p = 0.01), table 3.

**Table 3:** Total bile acid concentration in gallbladder bile collected from control mice or humanized mice, with or without injection of FGF19.

	Treatment	Sample	DCA	CDCA	AMCA	CA	UDCA	HDCA	BMCA	OMCA	Ratio		
		No.	%	%	%	%	%	%	%	%	DCA/ BMCA	Total ng/ul	Average
Humanized	FGF19	1	0.2	1.9	4.8	42.0	1.3	1.0	41.6	7.2	0.01	7431	9003
Humanized	FGF19	2	0.9	2.9	7.2	21.0	2.2	0.7	55.3	9.9	0.02	10986	
Humanized	FGF19	3	0.0	0.4	0.2	90.7	0.1	0.9	3.6	4.0	0.00	8593	
Humanized		4	0.6	6.5	9.3	32.6	4.4	0.5	29.4	16.7	0.02	19065	24517
Humanized		5	7.1	3.8	2.9	78.6	1.8	0.3	4.2	1.4	1.72	27861	
Humanized		6	17.4	3.0	3.1	66.6	2.5	0.3	3.8	3.3	4.58	26626	
Mouse	FGF19	7	0.6	0.9	2.4	30.0	5.1	1.4	21.7	38.0	0.03	9149	9454
Mouse	FGF19	8	0.8	0.9	2.5	30.7	4.9	1.4	22.8	36.0	0.04	9228	
Mouse	FGF19	9	0.9	0.9	2.4	30.5	5.3	1.2	22.1	36.8	0.04	9984	
Mouse		10	0.5	0.8	3.8	45.7	0.8	0.5	39.2	8.7	0.01	16495	17289
Mouse		11	0.0	0.4	1.9	73.2	0.3	0.6	19.1	4.5	0.00	13552	
Mouse		12	0.9	0.9	4.1	42.5	1.2	0.4	40.2	9.8	0.02	21819	

Statistics were performed on logtransformed data inorder to stabilize variances.

prior to one-way ANOVA followed by post-hoc analysis according to the least.

significance differance (LSD) test. In humanized mice the average bile acid levels was significantly lower after injection of FGF19, p = 0.001 and also in wild type mice bile acid levels was lower after injection of FGF19, p = 0.01. The overall significance of the experiment was p = 0.0048.

**Figure 3:**
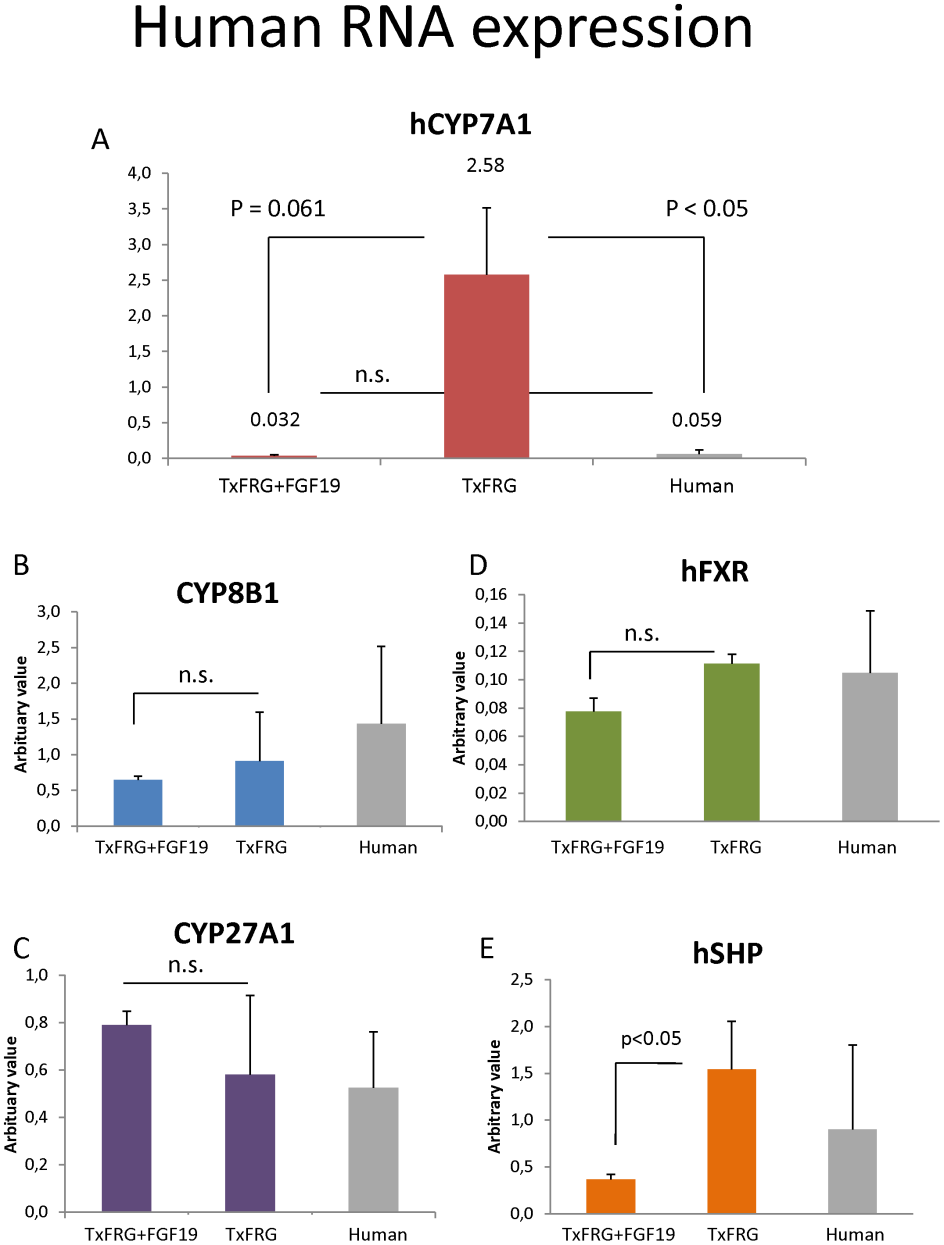
Expression of human RNA. A, Expression of human *CYP7A1* in humanized FRG mice (TxFRG) treated with FGF19 (TxFRG+FGF19) compared to human control. Statistics were performed by a non-parametric Kruskal-Wallis ANOVA. The overall significance of the experiment was p<0.05. Expression of human *CYP8B1* (B), *CYP27A1*(C), FXR (D) and SHP (E) in livers of humanized mice (TxFRG) treated with FGF19 (TxFRG+FGF19). Human liver RNA was used for reference (n = 9).

Expression of human *CYP7A1* was significantly (∼ 80-fold) decreased in humanized mice treated with FGF19 compared to controls, from 2.58 (arbitrary value) in transplanted FRGN, to 0.032 following FGF19 injection (p = 0.061). The expression of *CYP7A1* was not significantly different between FGF19 treated FRG mice and human controls, figure 3A.

**Figure 4:**
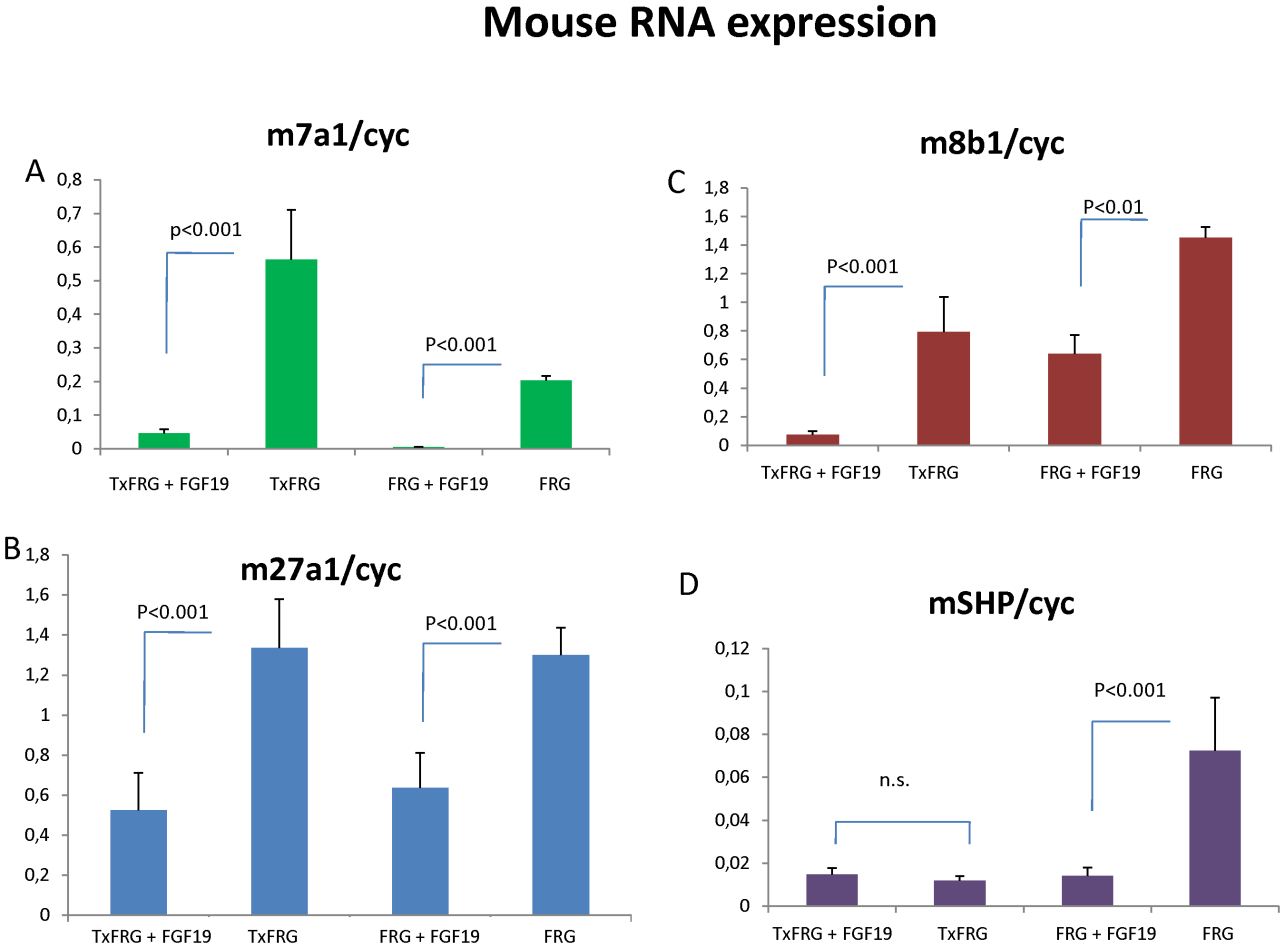
Expression of mouse RNA. A, *Cyp7a1*, B,*Cyp27a1*, C, *Cyp8b1*, and E, SHP in livers of both FRG and humanized FRGN mice (TxFRG), with or without FGF19 (TxFRG+FGF19, FRG+FGF19). Statistics were performed by a 1-way ANOVA on log-transformed data followed by LSD test.

RNA expression of *hCYP8B1*, *hCYP27A1* and the nuclear receptors, short heterodimer partner, SHP and farnesoid x receptor protein, FXR are shown in figure 3B-E. Expression of *hCYP8B1,hCYP27A1* and hFXR were not altered by administration of FGF19, however hSHP was significantly decreased (p<0.05),figure 3E. Administration of FGF19 significantly decreased mouse *Cyp7a1* (p = 0.001) expression in both humanized and non-transplanted FRG mice (n = 3) as expected (figure 4A). Expression of *mCyp8b1* and *mCyp27a1* were also significantly decreased by FGF19 injection whereas mouse SHP did not decrease in humanized mice, but significantly (p<0.001) decreased in the non-transplanted mice (figure 4B-D).

## Discussion

The lack of a small animal model of hepatic lipoprotein metabolism has limited research in this important area of biomedicine. Rodent and humans have cardinal differences in cholesterol metabolism and lipoprotein profiles that protect rodents from atherosclerosis. One of the major differences is the ratio of LDL and HDL and the cholesterol levels. Mice have lower levels of total cholesterol, and the major lipoprotein is HDL.

In this study we attempt to recreate human lipoprotein and bile acid metabolism in mice using FRG mice transplanted with human hepatocytes. Chimeric mice highly repopulated with human hepatocytes showed a shift from a HDL phenotype to a LDL centric distribution of lipoproteins. Mice with highly humanized livers showed lipoprotein profiles nearly identical to human plasma samples. Hence this mouse model will be an important tool to test the effects of drugs and gene therapy on the synthesis, secretion and uptake of human lipoproteins by hepatocytes. Moreover, in contrast to humans, rodents fed a high-cholesterol diet are resistant to the development of hypercholesterolemia [20], [21]. With the changes in lipoprotein levels observed in repopulated FRG animals, these mice may be sensitive to dietary cholesterol challenges. Additional studies are needed to test this hypothesis. Another important feature of the model presented here is the expression of human apolipoproteins, such as Apo E. Not only could we detect human Apo E (figure 1C), we could also discriminate different protein isoforms (not shown) from different cell donors. This is important because different phenotypes are associated with certain characteristics, for example ApoE2/2 is associated with type 3 dyslipidemia.

Bile acid amidation differs between species; mice conjugate almost exclusively with taurine whereas humans conjugate with both glycine and taurine at a ratio of approximately 5:1. We expected the conjugation pattern to be altered in humanized mice and we did see the appearance of glycine-conjugated bile acids in highly repopulated mice. The highest degree of glycine conjugation was on cholic acid (table 1). Unexpectedly we observed up to 11% unconjugated cholic acid in bile. This is puzzling, as the non-transplanted mice do not have much free cholic acid in bile. Free biliary bile acids, mainly cholic acid, have been described in rats (10–15%) and the possum. It is unusual to find free bile acids in bile of humans. Matoba et al reported that 0.1–0.4% of bile acids in bile were unconjugated, but these were mainly unusual C_23_ and C_27_ bile acids [22]. The occurrence of free cholic acid in highly repopulated mice observed here could simply be due a hepatic depletion of taurine. This hypothesis will be tested in future experiments by supplementation of dietary taurine.

We hypothesized that repopulation with human hepatocytes would also significantly alter bile acid composition. Table 2 shows the percentage of individual bile acids as well as the level of humanization and human serum albumin levels. The percentage of beta - muricholic acid (BMCA) is slightly lower in repopulated mice compared to non-transplanted FRG mice. Deoxycholic acid (DCA) also increased in humanized mice as expected, and the ratio of DCA/BMCA was significantly higher in both highly and moderately repopulated mice. It is somewhat surprising that high levels of repopulation (>80%) did not give a more completely humanized bile acid composition. This may be explained by the higher synthesis rate of bile acids in mice. Healthy humans synthesize about 500 mg of bile per day [23], which corresponds to about 0.35 mg per gram of liver. Mice synthesize 4.3 mg per day per 100 grams of body weight, which corresponds to about 0.78 mg of bile per day per gram liver [24], [25]. This rate is twice of that observed in humans and may contribute to the murine composition as would the ability of rodent hepatocytes to rapidly transform CDCA, DCA and CA into beta-muricholic acid [26].

The regulation of bile acid synthesis involves a complex series of events involving both the liver and intestines. Hepatic bile acid synthesis is feedback inhibited by bile acids returning to the liver via enterohepatic circulation. Thus, bile acid synthesis is stimulated if bile acids are constantly removed (via fistula) or inhibited by normal enterohepatic recirculation of bile acids or as in the case of drug therapy, where taurine conjugated bile acids are introduced into the intestines. A humanized mouse model offers a unique opportunity to examine the regulation of human *CYP7A1* and bile acids production *in vivo* and to investigate feedback signaling between the intestines and liver. In mice, FGF15, and in humans, FGF19, is thought to be released from intestines when bile acid pools are sufficient to inhibit the expression of *CYP7A1*, the rate-limiting step in bile acid synthesis in hepatocytes. We observe a 57-fold increase in the RNA levels of the rate-limiting enzyme *CYP7A1* in human hepatocytes in humanized mice as compared to normal human hepatocytes. We speculate that this is due to abnormal FGF signaling between murine intestine and human liver cells. Therefore, FGF19 was administered (s.q) in single or repeated injections and human (h) CYP7A expression and bile acids production was examined. As expected, FGF19 injection was sensed by the human hepatocytes and led to a dramatic decrease in both *hCYP7A* expression and bile acid production in the animals, confirming the hypothesis that lack of FGF19 lead to an increased hCYP7A expression and bile acid production. The positive response in human hepatocytes to FGF19 administration confirms that the human hepatocytes within the mouse liver respond to the species appropriate FGF with the expected outcome of suppression of CYP7A and bile acid production. This humanized FRG model offers a unique opportunity to examine human relevant modulation of bile acid production, *in vivo*.

The bile acid concentration in gallbladder bile was reduced following injection of FGF19 in both repopulated and control mice. The concentration of DCA was lower following injection of FGF19 in humanized mice whereas omega muricholic acid increased following administration in non-transplanted FRG mice. In repopulated mice injection of FGF19 leads to repression and a normalization of *hCYP7A1*. *hCYP8B1* was also repressed whereas *hCYP27A1* was not altered. However, hSHP expression did not increase following FGF19 injection, in fact it decreased. Holt et al. [27] suggested that FGF19 represses *CYP7A1* through a SHP independent mechanism. We previously reported that treatment with bile acids or FGF19 substantially increased SHP protein stability in cultured human hepatocytes or mice *in vivo* [28]. Therefore, the role of SHP in the regulation of *CYP7A1* by FGF19 remains unclear.

Our studies confirm previous studies that FGF19 down regulates mouse *cyp7a1*, in both control mice and humanized mice [27]. Interestingly, mouse Shp was down regulated by infusion of FGF19 in FRG controls, but not in repopulated FRG mice, however levels are already low in the repopulated mice and there was no further down regulation by FGF19 injection. One possible explanation for this could be that human hepatocytes subjected to high levels of bile acids in the FRG mouse express and secrete FGF19 in a paracrine manner and it has been suggested that human hepatocytes may contribute to the circulating FGF19 levels found in humans [29]. However, due to restricted amounts of serum available from these mice, analysis of circulating FGF19 levels could not be completed in the present studies.

## Conclusion

In this report we demonstrate that FRG mice repopulated with primary human hepatocytes display a serum lipoprotein profile nearly identical to humans including human apolipoproteins. Gallbladder bile of highly repopulated are altered towards a more human composition including the appearance of glycine conjugated bile acids. Also, increased levels of the secondary bile acid deoxycholic acid show that repopulated mice have a functioning enterohepatic circulation. Taken together, these results demonstrate that repopulated FRG mice have the potential to be a unique small animal model of atherosclerosis and cholesterol metabolism where not only the lipoproteins and bile acids are humanized, but the whole arsenal of functions that liver cells perform, including drug metabolizing enzyme systems. Our experiments with FGF19 injection also illustrate how this unique model can be used to elucidate regulatory pathways and the contributions of different organs to liver homeostasis.
