# Correction: Mice with Chimeric Livers Are an Improved Model for Human Lipoprotein Metabolism

**DOI:** 10.1371/journal.pone.0103870

**Published:** 2014-07-23

**Authors:** The PLOS ONE Staff

The second author’s name is incorrect. The correct name is Willscott Edward Naugler. The correct citation is: Ellis ECS, Naugler WE, Parini P, Mörk L-M, Jorns C, et al. (2013) Mice with Chimeric Livers Are an Improved Model for Human Lipoprotein Metabolism. PLoS ONE 8(11): e78550. doi:10.1371/journal.pone.0078550
